# “We Are Doing the Absolute Most That We Can, and No One Is Listening”: Barriers and Facilitators to Health Literacy within Transgender and Nonbinary Communities

**DOI:** 10.3390/ijerph19031229

**Published:** 2022-01-22

**Authors:** C. Riley Hostetter, Jarrod Call, Donald R. Gerke, Brendon T. Holloway, N. Eugene Walls, Jennifer C. Greenfield

**Affiliations:** Graduate School of Social Work, University of Denver, Denver, CO 80208, USA; jarrod.call@du.edu (J.C.); donny.gerke@du.edu (D.R.G.); brendon.holloway@du.edu (B.T.H.); eugene.walls@du.edu (N.E.W.); jennifer.greenfield@du.edu (J.C.G.)

**Keywords:** health literacy, transgender and nonbinary health, barriers to care, facilitators to care, healthcare

## Abstract

Transgender and nonbinary (TNB) individuals face disparities in nearly every aspect of health. One factor associated with poor health outcomes in other marginalized populations is health literacy, yet no identified studies examine health literacy in TNB samples. Moreover, most health literacy frameworks focus primarily on the capacities of individual patients to understand and use healthcare information, with little attention given to provider literacy and environmental factors. In partnership with a statewide LGBTQ advocacy organization, we recruited 46 transgender and nonbinary individuals to participate in seven focus groups conducted in urban, suburban, and rural locations throughout Colorado. TNB participants consistently engaged in efforts to increase their own health literacy and that of their medical providers yet faced multiple barriers to improve care. Difficulty identifying and physically reaching care, insurance and out-of-pocket expenses, negative experiences with healthcare providers and staff, provider incompetence, discriminatory and oppressive practices, and exclusionary forms and processes emerged as barriers to enacted health literacy among participants. Conversely, facilitators of enacted healthcare literacy included positive experiences with healthcare providers and staff, and inclusive forms and processes.

## 1. Introduction

Transgender and nonbinary (TNB) people are individuals whose gender does not match their sex assigned at birth and includes people who identify as men/masculine, women/feminine, as well as identities outside of the gender binary such as nonbinary, genderqueer, bigender, or agender [1]. As of 2016, there were approximately 1.4 million people (around 0.6% of the population) in the United States who identified as TNB [2], and this is likely an undercount resulting from high levels of discrimination and violence directed toward TNB people.

TNB people experience substantial barriers accessing appropriate healthcare [3,4], including having doctors refuse to treat them due to their gender [3], experiencing non-affirming or discriminatory care [5], encountering difficulty getting insurance to cover medically necessary procedures [6], and needing to educate providers who have not received training on TNB health [7,8]. These healthcare access barriers exacerbate already significant health disparities TNB people experience as a result of the systemic and structural forces of transphobia and cisgenderism [9,10]. For example, TNB people have higher rates of diabetes [11], heart disease [12], substance abuse, HIV, and self-harm [7] than cisgender people. Research on poor health outcomes in other marginalized populations suggests that increasing patient health literacy can help reduce barriers to healthcare and ultimately health disparities [13]. However, health literacy in TNB populations has been absent from the literature. As such, this study examines barriers and facilitators to healthcare access and health literacy among a statewide sample of TNB individuals in Colorado.

### 1.1. Barriers to Accessing Healthcare

Transgender and nonbinary individuals face numerous barriers in accessing healthcare, many of which are systemic [9]. These individuals frequently encounter discriminatory practices (e.g., denial of care, harassment) in response to their gender identities and expressions [14], resulting in transgender individuals opting to not seek care when they are sick at a rate that is twice as high as the overall U.S. population [5,7]. Among TNB people, nonbinary persons note greater amounts of time that pass with unmet medical needs compared to both transgender men and women as well as cisgender men and women [15]. Accessing affirming and TNB-inclusive care is another barrier for TNB individuals, and there is a lack of adequately trained providers [16,17,18]. Even once affirming care has been identified, TNB individuals may need to travel great distances and across state lines to receive care [8,19].

When TNB people are able to access care, many report non-affirming practices such as being misgendered, being deadnamed (i.e., referring to a TNB person by their given or birth name as opposed to their chosen name), or having incorrect pronouns used [20]. Both misgendering and deadnaming can cause significant distress for many TNB people [21]. Many of the forms used in healthcare settings also pose difficulties, in that patients must determine whether sex or gender is being asked and which to select, as well as complications regarding legal or birth names versus current names [22]. Furthermore, many TNB individuals experience high medical costs and inadequate insurance coverage for their healthcare needs [6,23]. Though some of this care is related to gender-affirming services including hormone replacement therapy (HRT), gender-affirming surgeries, or other transition-related care [6], TNB persons are also denied other services unrelated to transition, such as reproductive care [24].

TNB individuals also experience outright denial of care because they are TNB [7]. This occurs with general medical needs, care related to gender-affirmation, as well as mental health care [3,25]. TNB persons experience denial of their healthcare needs at significantly higher rates than cisgender lesbian, gay, and bisexual (LGB) individuals [10]. Taken together, barriers such as healthcare denials, limited access to affirming care, and potential discrimination result in TNB adults utilizing healthcare services at lower rates than their cisgender peers [25,26].

### 1.2. Facilitators to Accessing Healthcare

Research also identifies facilitators to healthcare access among TNB people, though this literature is less developed. One consistently discussed facilitator is fostering a TNB-inclusive environment by intentionally using correct patient names and pronouns [27,28], as well as including TNB-affirming language in advertisement and patient-facing materials [8]. TNB identities and people are often erased from healthcare settings that do not engage in these inclusive practices due to cisnormativity [9], and greater TNB representation fosters increased safety and comfort.

A second commonly described facilitator is support navigating complex medical systems primarily constructed around binary cisgender conceptualizations of gender. Specifically, this can be accomplished by providing patient advocates [24], integrating primary and specialty services into a single location [29], and coordinating between providers to prevent patients from having to repeatedly disclose their gender [30]. A third identified facilitator is appropriately trained providers who have experience working with TNB people. Patients who reported seeing a provider who was knowledgeable about TNB people, respected privacy, and asked questions were more likely to access healthcare in the future [27,30]. Social support is an additional facilitator to healthcare access. Specifically, TNB adults report that having the support of other TNB people made it easier to access care [24] and TNB youth who report higher levels of connectedness to adults accessed healthcare at higher rates [31]. Finally, research identifies financial stability as an important facilitator to accessing care. TNB people with health insurance [23] and those making over $20,000 a year [5] accessed healthcare at higher rates than those who were uninsured or made less than $20,000.

### 1.3. Health Literacy among TNB Individuals

Defined as an individual’s ability to seek, understand, and use healthcare information, health literacy has been shown to be associated with multiple healthcare outcomes, including healthcare access [32]. Individuals from marginalized communities who experience disproportionately negative health outcomes, such as racial or ethnic minorities, and low income populations, report lower levels of health literacy than those from less marginalized communities [33]. Accordingly, scholars have posited that increasing health literacy in marginalized communities may lead to reduced health disparities [13]. However, despite poorer healthcare access and health outcomes among TNB individuals, research has yet to address health literacy in this population.

The extant literature exploring barriers and facilitators of healthcare access for TNB individuals is growing, though there is still need for additional research. Most TNB research uses one of two national samples: the National Transgender Discrimination Study (NTDS) [7] or the U.S. Transgender Survey (USTS) [3]. These national datasets provide critical perspectives on the U.S. TNB population generally, but do not fully account for unique regional barriers and facilitators to care. Furthermore, almost all state-level research about TNB healthcare access has taken place in the eastern half of the United States, excluding large portions of the country [10]. These overlooked portions may have lower population density, including density of healthcare providers, and healthcare access may be fundamentally different from those in high-density coastal regions. Moreover, relatively few studies include the perspectives of nonbinary people, a population that is often overlooked despite making up one-third of USTS respondents [3]. Finally, research has failed to examine health literacy in TNB people. To address these gaps, our study seeks to identify the primary barriers and facilitators to healthcare access and health literacy among a sample of transgender and nonbinary Coloradans. Colorado represents an interesting area of study due to its mix of urban and rural regions, as well as sociopolitical diversity.

## 2. Materials and Methods

### 2.1. Positionality

Our research team had nine individuals with diverse gender identities and sexualities, with the entire team identifying as LGBTQ. The team was a combination of senior faculty, junior faculty, and doctoral and MSW students from the U.S., with all team members identifying as White and holding U.S. citizenship. We come together as individuals who advocate for TNB communities, with some team members identifying as TNB and having to interact with the healthcare system. As a team, we are committed to learning from the communities we research and amplifying their stories and lived experiences in a way that promotes social justice and brings awareness to both their positive and unjust healthcare experiences.

### 2.2. Theoretical Framework

A majority of health literacy frameworks focus primarily on the abilities of individual patients to understand and use healthcare information. Although provider knowledge and environmental factors may also be important to consider, these elements are seldom found in extant health literacy frameworks. Moreover, results of previous research indicate that TNB individuals may need higher levels of health literacy to compensate for lack of provider knowledge and training regarding TNB health and healthcare needs [34]. As such, we assert that extant health literacy frameworks are inadequate and not well suited for understanding the interactions of TNB individuals with the healthcare system.

**Figure 1:**
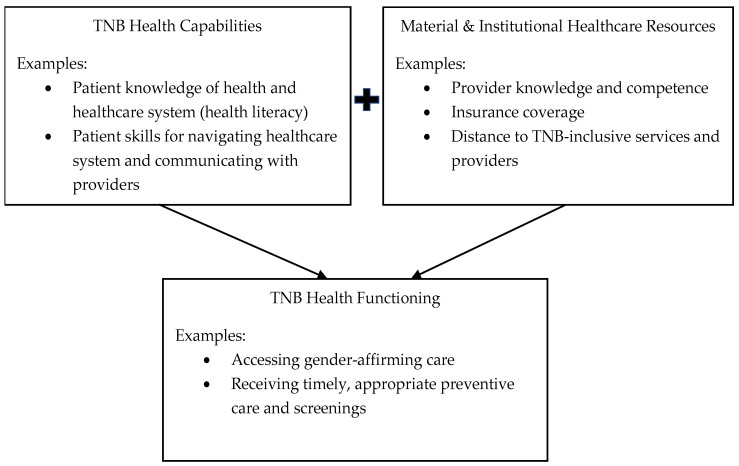
Capabilities framework of TNB health functioning.

Looking outside existing health-focused theoretical frameworks, we identified the capabilities approach, popularized by Sen [35] in the field of economics, as a model with promise for explaining how patient knowledge and the structural environment are both necessary and interacting aspects of access to appropriate and sufficient care within the healthcare system. In adapting the capabilities approach as an explanatory model of TNB health literacy/healthcare access, we conceptualize patient “capabilities” as those aspects of health literacy that are typically captured in prior models (e.g., patient knowledge & skills); the material/institutional “resources,” including provider knowledge and insurance coverage, is the second, equally necessary aspect of the full model. Together, these two interdependent and interacting components form the “functioning” of health literacy for TNB people. For instance, a patient’s “capability,” knowledge of hormonal treatment options, may be irrelevant if their “resources,” such as insurance coverage, do not include payments for those treatments. Thus, both the capabilities and resources are necessary for full functioning in the healthcare system. A primary aim of this study was to understand to what extent individual capabilities and structural/environmental resources act as barriers or facilitators for TNB people accessing healthcare, in order to explore how well the adapted capabilities approach aligns with TNB experiences. A visualization of this theoretical framework in shown in Figure 1.

### 2.3. Methodological Approach

#### 2.3.1. Study Sample

Participants were 46 self-identified TNB individuals living in the state of Colorado. The age of participants ranged from 19–72 years (M = 39.52, SD = 16.60) and the majority (n = 34) self-reported identifying as White/Caucasian. Other racial/ethnic identities self-reported by participants included Hispanic (n = 3), Asian (n = 3), Native American (n = 1), and more than one race/ethnicity (n = 5). When asked their gender identity, 23 participants (50%) reported being transfeminine, 12 (26%) reported being transmasculine, 9 (20%) reported being nonbinary, and 2 (4%) reported another gender identity. Sexual orientations reported by participants included bisexual/pansexual (n = 20), lesbian (n = 9), straight/heterosexual (n = 7), queer (n = 6), asexual (n = 2), gay (n = 1), and questioning (n = 1). Participant demographics are shown in Table 1.

#### 2.3.2. Recruitment & Data Collection

Focus group participants were recruited by emails from a statewide LGBTQ advocacy organization, through posts on social media (e.g., Facebook, Instagram), through fliers distributed to key stakeholders around the state, and through word of mouth. Fliers requested transgender and nonbinary people to participate in research to help improve health care for TNB individuals in the state. Interested individuals emailed or called the project PI (DRG) or scanned a QR code that led them to a HIPPA-secure online interest survey hosted on REDCap. After they completed this survey, a member of the project team contacted them to discuss the study and schedule their participation. To be eligible to participate, individuals had to identify as transgender, nonbinary, or gender diverse, be 18 years of age or older, and live in the state of Colorado. Our community partner agency was selected because they are Colorado’s largest LGBTQ advocacy organization and because the PIs have a history of engaging in research with the organization. Data were collected from seven focus groups held at locations across the state of Colorado, including Denver, Aurora (a suburb of Denver), Colorado Springs, Fort Collins, Durango, and Grand Junction. In-person focus groups were conducted throughout the summer of 2019. The seventh focus group was held online at the start of 2020 to accommodate those who wished to participate but were unable to attend any in-person groups.

The focus group guide included five primary questions that were asked of participants regarding their experiences finding and using healthcare information and services. Specifically, participants were asked where they found healthcare information, what they wished providers knew in order to provide quality healthcare services to TNB individuals, what an ideal healthcare system designed to serve TNB individuals would include, what impact their experiences with healthcare providers and systems as TNB individuals has had on their health, and what would help TNB individuals more effectively navigate the healthcare system. Focus groups lasted approximately 90 min and were audio recorded. Recordings were later transcribed by MSW student research assistants and verified by the primary investigator. Focus group participants each received a $20 gift card for their participation. Participants were also offered the opportunity to participate in member-checking prior to publication of results.

This study was approved by the Institutional Review Board at the University of Denver.

### 2.4. Data Analysis

Data analysis was conducted by a group of five LGBTQ individuals who were part of a larger research team. This group was a subset of a larger team of seven coders, all of whom identify as LGBTQ. Each transcript was coded in Dedoose by at least two team members. A foundational round of in vivo coding [36] was first conducted until saturation was reached. These codes were utilized for an initial codebook, which was then reviewed by PhD-level research assistants for trustworthiness and to further the thematic coding process. Any discrepancies in the coding process were resolved as a team via a consensus process.

Following in vivo coding, thematic analysis was done to identify patterns and themes in the transcripts. The data analysis team reviewed all themes again to verify and corroborate that all concepts had been included. Meaning-making [37] was then conducted by the team, which included further exploring primary codes and sub-codes to gain a more thorough understanding of the themes and codes, as well as identifying salient quotes and context. The meaning-making document was further used for member-checking by focus group participants who expressed enthusiasm for the results and identified no substantial concerns. Lastly, the focus group transcripts were again reviewed by a smaller subset of three team members focusing on the barriers and facilitators noted, as well as to synthesize material into discernible components.

## 3. Results

We identified two themes to capture our findings: (1) barriers to accessing care, and (2) facilitators to accessing care. Categories for barriers to accessing care include (1) difficulty identifying and physically reaching care, (2) finances and insurance, (3) negative experiences with healthcare providers and staff, and (4) exclusionary forms and processes. Categories for facilitators to accessing care were (1) positive experiences with healthcare providers and staff and (2) inclusive forms and processes. These themes highlight some of the ways in which institutional resources can impact one’s ability to access appropriate care despite their own health capabilities and knowledge.

### 3.1. Barriers to Accessing Care

#### 3.1.1. “We Need More Ways to Access Care”: Difficulty Identifying and Physically Reaching Care

Participants from all focus groups highlighted examples of difficulty identifying TNB-competent care and difficulties physically accessing care. For participants who found it difficult to identify TNB-competent care, one barrier was resources and information about TNB healthcare being hard to find, including on insurance company websites. This resulted in one participant having to call 60 medical providers to find a provider who would write a referral letter for a gender-affirming surgery:

They [insurance company] don’t have a list of doctors who are covered and write letters for surgeries. So, I had to call about 60 doctors. And out of that, maybe 2 of them—well, 2 of them said they’d give it a try, but they had no experience.

Additionally, some participants who did find information about TNB healthcare online found that some information was inaccurate, unreliable, or outdated. They explained, “Just doing a Google search can sometimes be trepidatious. You don’t always get good information, or you get lots of diverse information and it’s not really a lot of good trustworthy sources.” Many participants who found reliable information online credited other TNB people for sharing the most accurate, helpful information.

Participants from all focus groups gave examples of how it can be difficult to physically reach care. Some reasons noted for this were not having reliable transportation, needing to travel far distances, and experiencing long wait times to see a TNB-affirming provider. To help individuals reach care, one participant proposed having more options:

I know people that drive like 5 hours to get to care. And so, I think having more available options, whether that’s virtual care where you can interface with the doctor via video or texting, having that available where you don’t always have to go to a physical clinic. We need more ways to access care.

Another barrier to reaching care for some participants was long wait times to see an affirming and competent provider for both general and transition-related healthcare needs. One participant described being told by a patient scheduler that, “The wait list is… ‘you’re gonna be lucky if you get in in three years.’” Moreover, insurance companies exacerbated already extensive wait times for some participants due to extended appeals processes after initial insurance denials. This was most prevalent for “gendered” procedures, such as mammograms and pap smears, as the insurance companies would not approve care for individuals with gender markers they perceived to be mismatched with the procedures.

Lastly, some participants noted isolation as a reason for not being able to reach care. Isolation was defined in two different ways by participants: some participants experienced loneliness from being misunderstood by others when attempting to navigate care, discouraging them from further seeking out services. Other participants described harmful policies that isolated them from cisgender people in inpatient settings, forcing them to wait longer than cisgender people because the facilities did not allow TNB patients to share treatment rooms with cisgender patients.

#### 3.1.2. “We’re Largely Powerless”: Insurance and Out-of-Pocket Expenses

A second barrier to accessing care reported by TNB individuals was health insurance. Some participants reported not having insurance, and even when participants were insured, their plans often did not cover gender -affirming care. For one participant who had insurance, their insurance provider unexpectedly added a deductible while they were in the process of receiving transition-related electrolysis care:

I think we’re largely powerless in many ways. The electrolysis was mentioned earlier, and my insurance was covering it with no deductible, and I’ve just been informed informally, never officially, that now I have a deductible that could amount to fifty percent payment, so drastically altering what I have available.

Another participant discussed a similar issue in relation to phalloplasty surgery. This participant had health insurance through their employer, which created a barrier because they were required to stay with their employer for the duration of their surgeries due to the insurance coverage:

When you’re talking about surgeries spread out over a long period of time, especially, and even if you don’t have complications with phalloplasty, you’re looking at around a year to two years, somewhere in there. If you have complications, it can go way longer than that, so the problem is, then, that it keeps you in employment, assuming that you got this far, you’ve got employment, you have health insurance at work. You’re stuck there.

For many participants with and without insurance, out-of-pocket expenses were a notable barrier to accessing care. In addition to high out-of-pocket costs for physical healthcare, psychotherapy is often required for TNB individuals who pursue transition-related procedures due to guidelines by the World Professional Association for Transgender Health (WPATH) [38] and general gatekeeping from providers. One participant highlighted how the additional expense for therapy further exacerbated the high cost of care: “Therapy is incredibly expensive, just because I see my therapist, she’s $145 a session completely out of network, non-reimbursable.” Overall, many participants voiced their concerns about out-of-pocket medical expenses and how these expenses often prevent them from accessing needed transition-related care.

#### 3.1.3. “Why Am I Supposed to Trust You to Treat Me?”: Negative Experiences with Healthcare Providers and Staff

Another frequently described barrier to care was past negative experience with healthcare providers. This showed up in multiple ways including encountering providers who were incompetent regarding TNB health and healthcare, as well as experiencing discriminatory and oppressive practices in healthcare settings. Participants shared concerns about receiving improper care both because their providers were not knowledgeable enough on TNB-related care, but also because some providers focused solely on their TNB identities.

##### Provider Incompetence

Discussion about incidents with trans-incompetent providers were a common theme throughout the focus groups. Participants noted multiple situations in which their providers were lacking in knowledge about TNB health. This frequently included providers not understanding gender-affirming treatments, but also involved providers asking invasive and inappropriate questions (e.g., questions about genitalia when health concerns were unrelated). Participants also shared experiences with violations of confidentiality and being outed in their healthcare setting, both by providers, as well as other staff.

With a lack of knowledge from medical providers, many patients expressed feelings that they needed to educate their providers, which they described as “trans 101.” Many also expressed that even after providing explanations and answering questions, their provider still seemed unaware and often continued to ask the same questions multiple times. Other participants discussed how they started creating documents and sharing articles of standards of care to give to their providers in order to ensure they received appropriate care.

I saw my primary for a checkup, and they were like, “So, will we need to do mammograms as you get older?” And I’m like, “You should know…! Shouldn’t you be telling me?…” It’s almost like the doctors have more questions for us than questions that we have for doctors.

In addition to causing issues with gender-affirming care, the lack of provider understanding also interfered with other medical treatments. Participants discussed experiences where their providers were unaware of how gender-affirming treatments and medications could interact with other medical needs, such as diabetes care or birth control. The overall lack of knowledge by providers elicited a lack of trust with their patients, particularly when there was not another option to find a different, more knowledgeable, provider. One participant expressed frustration around this stating, “Why am I supposed to trust you to treat me if you don’t even know how, what’s going on in my body, is gonna interact with what you’re gonna do to me?”.

##### Discriminatory & Oppressive Practices

In addition to receiving poor care, or having providers that lacked adequate knowledge, other participants spoke of providers refusing them care because they were unfamiliar with gender-affirming care or did not feel comfortable treating TNB individuals. Furthermore, participants reported invasive and adverse experiences with providers giving the incorrect dose of medications, deadnaming patients, dissuading and encouraging various medical decisions and procedures, among others. Moreover, the significant lack of provider knowledge further exacerbated difficulties in ascertaining appropriate and reliable medical care.

Participants also spoke about concerns of their TNB identities distracting their provider from other relevant medical needs. One participant described their interaction with a primary care physician when attempting to access care unrelated to their gender:

I had one physician that I went into for just primary care stuff; I was sick. And was talking to them, and they were… completely uneducated on being transgender, and were asking me, “Why [I] made this choice,” and were really encouraging me to stop hormones, things like that. And so that was pretty difficult to deal with.

Participants also shared experiences in their care needs not being addressed because their providers solely focused on their transgender identities. For example, one participant stated, “It’s not rare where people just get so blindsided about our being trans or nonbinary, intersex, that what we’re there for goes away.” Others discussed experiences in religious-based healthcare settings and being outright denied care.

Numerous participants also discussed the experiences of managing unfriendly office staff, as well as staff that gave unsolicited advice and opinions. Office staff were most frequently named as personnel that deadnamed participants and broke confidentiality. One participant explained, “I think the other thing is even if the providers are really good, if the front office staff is not trained or bought into it, that’s hurtful”.

Nonbinary patients also expressed difficulty in engaging with healthcare due to its gendered nature, suggesting that “if you’re not binary, you don’t exist.” They reported both feeling pressured by medical providers to begin hormones in order to “fully” transition, while also having less opportunity to access hormones due to providers not understanding their identities. These additional barriers for nonbinary folx were described across focus groups and highlighted by one member located in Denver:

I will say for me that since my identity has shifted from nonbinary to more trans-femme that things have actually gotten simpler for me because for all those years that I identified as nonbinary and I was going by a gender-gray nonbinary name, it always felt like an uphill battle and very often I just gave up fighting, but I’ve found that since I changed my name to a more obviously female name that, you know, I have yet to be misgendered or misnamed by somebody who’s like paid to not to.

#### 3.1.4. “You Would Be Constantly Proving That You’re Trans Enough”: Exclusionary Forms & Processes

Finally, participants described exclusionary forms and processes as barriers to accessing healthcare. One way this showed up was in rigid intake forms that either did not have anywhere to indicate being transgender or only included binary options with no recognition of nonbinary or intersex people. This was described by a participant who shared, “I crossed everything out and wrote intersex in because I’m not an ‘other’ or any of the other identities listed there.” Many participants also expressed frustration with the need to include their legal name and sex assigned at birth on intake forms as required by healthcare and insurance organizations. Participants explained that having this information on file increased their likelihood of being deadnamed, as some providers referred to patients by legal birth names even when a preferred name was provided.

According to one participant: “I have my name and gender legally changed and everything, and I am still fighting with healthcare providers because their automated system will send me a message that deadnames me.” In addition to encountering non-affirming intake forms, participants described having providers and insurers automatically recommend treatments that did not make sense for their anatomies. This was described by one participant reporting, “I’ve changed my gender marker legally, and [my provider online portal] is like, ‘Hey, you need a pap smear and a pregnancy test.’ I’m going, ‘But in the other box you have transwoman selected, so why is all of this other crap popping up?’” For this participant and others, experiencing non-affirming and exclusionary forms led them to feel unsafe and unwanted from the beginning of their visit.

Participants also described how exclusionary forms operated in the context of insurance. Several participants were denied coverage for medically-necessary treatments, including hormone replacement therapy, because the treatments that supported their anatomies did not always align with insurance expectations based on legal gender markers. As one participant described, “There was a claim that was rejected thirty-two times I think, because…their sex is legally male, but they are having female issues…luckily, the provider is understanding with that, but it’s a year and a half of sitting there with a $185,000 bill over your head, and it’s not being covered.” Although some participants expressed that these insurance issues were eventually resolved, others continued to battle with forms and procedures designed around a cisnormative and binary perception of gender. In addition to experiencing these barriers in healthcare clinics, participants noted experiencing similar difficulties at pharmacies when attempting to access hormones. According to one participant:

Sometimes it’ll just be an automatic denial if the gender doesn’t match who [insurers] think should receive that treatment. So, if you’re labeled ‘M’ at the pharmacy you’re automatically denied from your insurance for estrogen. If you’re labeled ‘F,’ automatic denial for testosterone. There really should be more flexibility with pharmacies around choosing what it should say so that you can actually have it covered by your insurance.

Furthermore, appealing insurance denials consumed significant time and energy, even among participants who eventually successfully obtained reimbursement.

Additionally, participants described how the process of accessing gender-affirming treatments was often unnecessarily difficult, largely in part because healthcare providers and insurers have traditionally followed guidance by the WPATH Standards of Care. These suggest that patients receive a diagnosis of gender dysphoria (formerly Gender Identity Disorder) and a letter of support from a mental health professional prior to receiving gender-affirming treatments. In essence, mental health providers act as gatekeepers in deciding whether TNB patients can receive treatment. As one participant explained:

That was my number one concern, what I was really worried about when I started treatment was that it would be like basically a constant test, like the thing with the gatekeeping, that you would be constantly proving that you’re trans enough or that you’re feminine enough, or whatever the case may be, that the doctor would actually want to see you to approve treatment and I did get a little of that with the therapist I saw.

Another participant described the negative mental health effects of being denied access to gender-affirming medical care through the WPATH model:

I’m not gonna talk to a cis person about what’s going on in my brain…I was told that I had to come back after a year and sit and think about whether or not I wanted HRT because I was too depressed to transition. And it turns out, transitioning was what helped me not be depressed.

Participants overwhelmingly voiced a preference for an informed consent model [39], in which TNB patients work directly with their medical providers to choose treatment options after receiving information about the risks and benefits of each option without needing a mental health referral letter to access gender-affirming treatment. This preference was exemplified by one participant who responded to a question about what an ideal healthcare system designed to meet the needs of TNB patients would look like: “Abolishing WPATH? [laughter] I don’t know, but it seems like a place to start. Get rid of all gates.” There were, however, a small number of participants who stated that some gatekeeping is beneficial. As described by one participant:

That perfect system would be, I still think… I mean, call me ruthless, but I still think that there should be a little bit of gatekeeping in place for like, to help us think. I did four years of therapy before I decided to go on hormones, like, for me, I needed that, I needed to process that out.

### 3.2. Facilitators to Accessing Care

#### 3.2.1. “Respecting My Body”: Positive Experiences with Healthcare Providers and Staff

A significant contributor to participants accessing care and feeling like their needs were being met was having positive interactions with medical staff and providers. Participants expressed positive feelings and experiences when they felt as though they were being heard and respected, and the provider was invested in their care. Invested providers asked questions of participants about what it is they were wanting and needing, as opposed to making assumptions about what was best. Provider collaboration with their patients and taking initiative to learn more about transgender and nonbinary care were large factors in feelings of provider competence. As described by one participant:

That’s the thing that has to be debunked if we’re trying restructure this system. The second that somebody says, “I’m trans,” it’s not, “We’re gonna make you a man,” “Make you a woman,” or, “Make you that person,” “We’re going to help seek out the kind of person… you were always meant to physically show yourself as”.

Participants across focus groups discussed how critical it was to have a provider that worked collaboratively with their patients and was accepting of input and feedback. These experiences allowed participants to feel more empowered in their care, and that they could advocate for the care that would be most beneficial. These included components regarding medications as well as other treatments, whether gender related or not.

Participants also expressed how this collaborative approach was helpful in that they no longer felt like they had to have all the answers about their care, but instead had a practitioner who was educating themself.

Either you’re expected to be the expert, or you get gatekept and you have to, like, fight for what you need and for her, like with my physician, it feels very much like she treats me like a participant, like an informed participant, and she’s open to trying new things.

Other participants shared positive experiences about medical staff stating they would do research on a medication and get back to them, which was a novel experience. These experiences also opened the door to having more transparent conversations about treatment plans and patients being able to advocate for their needs in an affirming setting.

Feeling respected by medical staff and professionals was also key in participants having positive healthcare experiences. This included using correct names and pronouns, as well as having medical staff that valued participants’ perspectives and asked them questions to fully understand their holistic selves. Some participants spoke to how this moved past questions about treatments and medications, but also included questions regarding preferred language for body parts:

My current doctor asked me what I preferred for my genitals to be called, and that was really cool, and felt like pretty advanced, [scoffs] in this world. So, it was respecting my body and how I view my body. That was, that was nice.

Another participant spoke to feeling their whole self was respected when their medical office assisted them in getting information on how to legally change their name and gender marker, and how this made them feel accepted and respected. Participants also spoke to the importance of feeling their names and pronouns would be respected, with one reporting it was a primary reason in why they see their current provider: “It is great when the staff treat you the way you want to be treated. They’re aware. They get the pronouns. They know the language. That’s nice. It’s why I go to the doctor I go to”.

#### 3.2.2. “There’s Nothing Better about Pronouns than Having Someone Model It”: Inclusive Forms and Processes

Another consistently described facilitator to accessing affirming healthcare was inclusive forms and processes. In particular, participants expressed support for intake forms that were inclusive of their gender, names, and pronouns. According to one focus group participant:

Since I started transitioning, they’ve updated their forms throughout the local hospital to have your pronouns, and I think they have a slot for sex and gender identity. I don’t know what that was the result of, but it was exciting to see.

This was echoed by a participant who described similar improvements to forms over time:

My last OB/GYN appointment was a year ago and everything was gendered. I went back like a month ago and nothing was. The front desk didn’t gender anyone. There was nothing on paper I had to fill out that was sex or gender. It was completely different after only a year, and it was way better.

Even in healthcare settings that had not updated their forms or electronic medical records, participants expressed that healthcare providers can create work arounds to better affirm patient identities: “We have found a workaround where we’ll put the name a person goes by in the ‘nickname’ part of the demographics. The insurance does have to be billed under what’s on their insurance card, but we can put their name and pronouns there.” Healthcare providers who intentionally sought to affirm patient identities through inclusive forms contributed to feelings of safety, encouraging participants to access these services.

Participants also described how inclusive processes facilitated the use of healthcare services. In particular, multiple participants referenced Jude’s Law, a recently passed Colorado law that allows TNB Coloradans to update the gender on their birth certificate to male, female, or nonbinary without surgery, a doctor’s note, or court order. It also removed the requirement that name changes be published in a newspaper [40]. Increasing the ease with which participants were able to change their legal documentation eliminated key barriers to accessing care as described by one participant:

There’s a long process to getting IDs and stuff updated in the system, so I’m not changing any of my information until Jude’s Law goes into effect and I can get [my birth certificate, passport, and driver’s license] all lined up. Those all need to be in line. What happens if I’m denied care because they are not all in line?

Participants also expressed that having access to TNB-affirming patient navigators or ombudspersons made it easier to navigate cisnormative medical systems. During a discussion in one of the focus groups about how to find an affirming primary care provider one participant stated, “LGBT navigators really helped me with that… [a local medical center] has a couple of those on staff and they will find doctors who are knowledgeable about HRT and trans issues.” Although the concept of LGBT-specific navigators was universally praised, some participants expressed that these navigation programs were often understaffed, making it difficult to connect and increasing wait times. Similarly, participants shared that having an affirming advocate attend appointments with them relieved them of some stress associated with medical settings. For example, when asked about factors that helped them navigate healthcare systems one participant said, “An ombudsperson. Somebody who you can call to basically say, ‘Hey I’m going to go to this appointment, come with me.’ There is nothing better about pronouns than having someone model it.” Resources like LGBT navigators and ombudspersons eased some of the additional stress TNB people experience when identifying providers, facilitating increased healthcare access and use.

## 4. Discussion

Results from this study support a capabilities approach to understanding health literacy among TNB individuals, as well the impacts on healthcare access. Although TNB participants in this study self-reported high levels of the *capability* of health literacy (e.g., knowledge of gender-affirming treatments, skills to find healthcare information), many were still unable to access affirming care because of *resource* barriers in the material and institutional healthcare environments [35]. For example, low levels of provider knowledge of TNB health, difficulty locating affirming care, exclusionary forms, and financial and insurance difficulties limited the extent to which TNB individuals were able to use their health knowledge to receive quality care. Conversely, TNB individuals were able to meaningfully participate (i.e., enact their health literacy capability) in their own gender-affirming care when they were able to access knowledgeable and collaborative providers that used inclusive forms and language. Thus, unlike the experience of some other marginalized groups where increased health knowledge may lead to improved healthcare access, in the TNB population, the resource environment—including access to knowledgeable and affirming providers—was reported by participants as more a significant influence on their overall health capability.

Moreover, our findings align with previous work in the areas of healthcare access for TNB individuals, suggesting that an increase in access to care and capable providers may also increase one’s health capability [33]. The TNB individuals in these focus groups often reported holding a higher degree of health knowledge pertaining to TNB-specific care than their providers. However, despite the knowledge held by participants, they self-reported that their efforts to increase their own health literacy did not lead to an improvement in care, and further amplified the systemic issues at play.

Accessing affirming care is a consistently noted barrier for TNB individuals [20]. These findings support the extant literature around this, as well as emphasize facilitators to care, including the importance of not only TNB-inclusive and affirming providers, but overall processes and paperwork as well. The ability for TNB patients to feel adequately cared for is paramount, both from healthcare providers themselves, but also from other staff involved. The importance of correct use of names and pronouns, as well as the use of healthcare navigators previously noted is echoed amongst our participants [24,28]. Taken together, these findings suggest that decreasing health disparities among the TNB population requires a focus on improving the resource environment rather than simply seeking to increase patient health knowledge/health literacy. The “health capability” approach is therefore a useful framework for understanding the dynamics of both health literacy and healthcare access as interdependent components of overall health capability in the TNB population.

Recently, a growing number of healthcare centers are transitioning from a gatekeeping WPATH model to an informed consent model where TNB patients work directly with their medical providers to choose treatment options after receiving information about the risks and benefits of each option without needing a mental health referral letter to access gender-affirming treatment [41]. This represents a positive step in empowering TNB people to determine how and when they want to access healthcare, though most insurers still require a diagnosis to access surgical care [42]. Gatekeeping requirements such as requiring a therapist letter for insurance coverage add additional steps to an already long and costly process.

These findings also have implications for practice and policy. Healthcare workers in all settings work with TNB people and incorporating lessons from these focus group data will better enable providers to serve their TNB clients with dignity. For example, one commonly discussed facilitator was when service providers affirmed TNB identities by using correct names and pronouns, both on intake forms and in person-to-person interactions. Intentionally asking clients what language they feel most comfortable with and sharing personal pronouns can create a safe space that fosters client and provider relationships. Healthcare workers should both implement affirming care such as this in their personal practices, and advocate for policy changes in their agencies and communities to affirm TNB identities by including affirming language on forms, simplifying the process of applying for name and gender marker changes, providing service navigators to connect TNB people with affirming services, and ensuring that insurance policies include TNB health needs.

Participants also frequently highlighted the importance of provider education. Providers who work with TNB people, including social workers, physicians, and other health professionals should actively seek to educate themselves about TNB people and health. While this can and should happen on the individual provider level, it is also imperative that professional organizations such as the Council on Social Work Education (CSWE) and Liaison Committee on Medical Education (LCME) incorporate TNB curriculum into program requirements, ensuring that health service providers such as social workers and physicians are appropriately trained to support TNB people in their practices.

### Limitations

Results of this study must be interpreted in the context of the study’s limitations. Participants were primarily recruited through a nonprofit organization’s network. As a result, the study sample was limited to locations around the state of Colorado and findings may not be generalizable to TNB individuals living in other states or areas of the country. However, this is one of the first studies to describe healthcare access and health literacy among TNB individuals living in the Mountain West region of the U.S. The sample also predominantly self-identified as White/Caucasian, so the results may not accurately reflect the experiences of TNB individuals of color. Additionally, some of the negative experiences shared were explored as instances of provider incompetence, as well as discriminatory and oppressive practices. The distinctions between these two aspects need to be further explored in future research, as incompetence itself may be a subtle form of discriminatory and oppressive practice.

## 5. Conclusions

Focus groups (n = 7) across Colorado were completed with 46 TNB individuals to capture what barriers and facilitators TNB people experience when accessing healthcare as well as extent of health literacy. Barriers for TNB individuals when accessing healthcare included difficulty identifying and physically reaching care, finances and insurance, previous negative experiences with healthcare providers and staff, and forms and processes that were TNB-exclusionary. Facilitators to accessing healthcare included previous positive experiences with healthcare providers and staff, and forms and processes that were TNB-inclusive. Participants were asked about how they navigate healthcare and learn about their health needs, resulting in an additional focus on participants’ health literacy. Participants from the study noted needing to have higher levels of health literacy to navigate healthcare, although these higher levels of health literacy did not result in accessing healthcare more. This is likely a result of the systemic healthcare barriers mentioned above.

Healthcare practitioners can address some of these systemic barriers by advocating for TNB-inclusive insurance policy changes, supporting healthcare offices to update their forms to be more TNB-inclusive, and working with healthcare offices in more remote areas to help providers and staff understand TNB people and their healthcare needs. Health educators can address some of these barriers by incorporating TNB-specific content into their classroom curriculum, specifically in medical education settings. In a system that was not designed to support and care for TNB people, it is our responsibility as healthcare professionals to advocate for TNB individuals to make healthcare as TNB-inclusive as possible.

## Figures and Tables

**Table 1:** Study sample demographics (n = 46).

Demographic	n (%)
**Race & Ethnicity**	
White/Caucasian	34 (73.9)
Hispanic	3 (6.5)
Asian	3 (6.5)
Native American	1 (2.2)
More than one race/ethnicity	5 (10.9)
**Gender**	
Transfeminine/transgender woman	23 (50.0)
Transmasculine/transgender man	12 (26.1)
Nonbinary/Agender	9 (19.6)
Another gender identity	2 (4.3)
**Sexual Orientation**	
Bisexual/pansexual	20 (43.5)
Lesbian	9 (19.6)
Straight/Heterosexual	7 (15.2)
Queer	6 (13.0)
Asexual	2 (4.3)
Gay	1 (2.2)
Questioning	1 (2.2)
**Age**	39.5 (16.60) *

* M (SD).

## Data Availability

No applicable.
